# Inter-Rater Reliability of Provider Interpretations of Irritable Bowel Syndrome Food and Symptom Journals

**DOI:** 10.3390/jcm6110105

**Published:** 2017-11-04

**Authors:** Jasmine Zia, Chia-Fang Chung, Kaiyuan Xu, Yi Dong, Jeanette M. Schenk, Kevin Cain, Sean Munson, Margaret M. Heitkemper

**Affiliations:** 1 Division of Gastroenterology and Hepatology, Department of Medicine, University of Washington, Seattle, WA 98195, USA; 2 Department of Human Centered Design and Engineering, University of Washington, Seattle, WA 98195, USA; cfchung@uw.edu (C.-F.C.); xky1231@uw.edu (K.X.); dyellen@uw.edu (Y.D.); smunson@uw.edu (S.M.); 3 Fred Hutchinson Cancer Research Center, Seattle, WA, 98109, USA; jschenk@fredhutch.org; 4 Department of Biostatistics and Office of Nursing Research, University of Washington, Seattle, WA 98195, USA; cain@uw.edu; 5 Department of Biobehavioral Nursing and Health Informatics, University of Washington, Seattle, WA 98195, USA; heit@uw.edu

**Keywords:** irritable bowel syndrome, diet, self-management, journaling

## Abstract

There are currently no standardized methods for identifying trigger food(s) from irritable bowel syndrome (IBS) food and symptom journals. The primary aim of this study was to assess the inter-rater reliability of providers’ interpretations of IBS journals. A second aim was to describe whether these interpretations varied for each patient. Eight providers reviewed 17 IBS journals and rated how likely key food groups (fermentable oligo-di-monosaccharides and polyols, high-calorie, gluten, caffeine, high-fiber) were to trigger IBS symptoms for each patient. Agreement of trigger food ratings was calculated using Krippendorff’s α-reliability estimate. Providers were also asked to write down recommendations they would give to each patient. Estimates of agreement of trigger food likelihood ratings were poor (average α = 0.07). Most providers gave similar trigger food likelihood ratings for over half the food groups. Four providers gave the exact same written recommendation(s) (range 3–7) to over half the patients. Inter-rater reliability of provider interpretations of IBS food and symptom journals was poor. Providers favored certain trigger food likelihood ratings and written recommendations. This supports the need for a more standardized method for interpreting these journals and/or more rigorous techniques to accurately identify personalized IBS food triggers.

## 1. Introduction

Up to 65% of patients with irritable bowel syndrome (IBS) associate certain foods with their symptom flare-ups [1]. Common exclusionary diets include gluten-free, non-spicy, and decreased intake of fermentable oligo-, di-, mono-saccharides, and polyols (FODMAPs) [2,3,4,5]. Although an IBS patient can theoretically follow all known exclusionary diets (or elimination diets) for symptom reduction, this is neither feasible nor necessary. First, a strict elimination diet is difficult, if not impossible, for most patients to follow [6]. Elimination diets can lead to significant weight loss and nutritional deficiencies [1,7]. Second, such a restrictive diet is likely not necessary to achieve the maximum symptom reduction possible from dietary changes. This is based on the belief that IBS patients have “personalized trigger foods” where they self-identify different combinations of trigger foods [1,6,8,9].

Identifying personalized trigger foods is a commonly accepted IBS management strategy [10]. However, there is currently no standardized methodology for identifying personalized trigger food(s) from IBS food and symptom journals. In a study by Chung et al., 21 IBS providers had variable and vague responses on how they reviewed journals [11]. They reported “picking out typical and … random dates” to “come up with some intervention(s)” [11].

To our knowledge, the inter-rater reliability of providers’ interpretations of IBS journals has never been formally studied. Such information can affect the future use of IBS journals in clinical practice. It can also support changing our methods of identifying personalized trigger food(s) from these journals. If providers disagree widely on their journal interpretations, this questions the reliability of the current methods used to identify personalized trigger foods. This also emphasizes the need for a more standardized method of reviewing such journals and/or more rigorous techniques, such as trial diets (e.g., an elimination-challenge diet) to accurately identify personalized IBS food triggers. However, if providers agree highly with one another on their journal interpretations, this supports the ongoing use of the current variable methods used to identify personalized trigger foods.

Our primary aim was to assess the inter-rater reliability of providers’ interpretations of IBS food and symptom journals. Specifically, we assessed providers’ agreement of (1) trigger food likelihood ratings of the following food groups: FODMAPs, high-calorie, high-fat, gluten, caffeine, high-fiber, spicy foods, and alcohol; and (2) written recommendations for individual IBS patients. A secondary aim was to describe whether providers varied their journal interpretations from patient to patient. We also explored each provider’s method of interpreting these journals and their perspectives on the use of such journals in clinical practice.

## 2. Experimental Section

This is a descriptive pilot study using paper food and symptom journals collected from 17 IBS adults from a prior feasibility and usability study [12]. Providers were recruited through mailings at a single academic center (Seattle, WA, USA) and by word-of-mouth. To be eligible, providers had to (1) see at least one IBS patient a month, (2) be familiar with the dietary management of IBS, and (3) review at least 10 journals during his/her career (one within the past three months). The study was approved by the Institutional Review Board from July 2015 through December 2015. Written, informed consent was obtained from each patient included in the study. The study protocol conforms to the ethical guidelines of the 1975 Declaration of Helsinki as reflected in a priori approval by our institution’s human research committee.

### 2.1. Provider Perspectives

**Figure 1:**
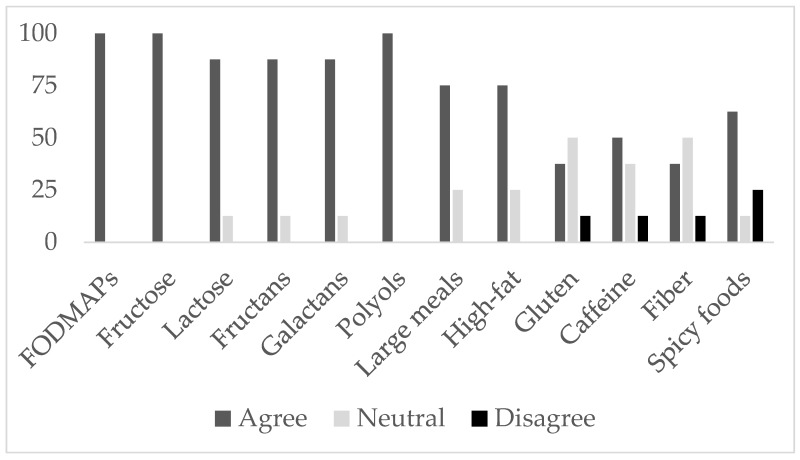
Intake questionnaire: provider perspectives prior to the study on whether they agree or disagree that select food groups are potential irritable bowel syndrome triggers. FODMAPs = fermentable oligo-di-monosaccharides and polyols.

**Figure 2:**
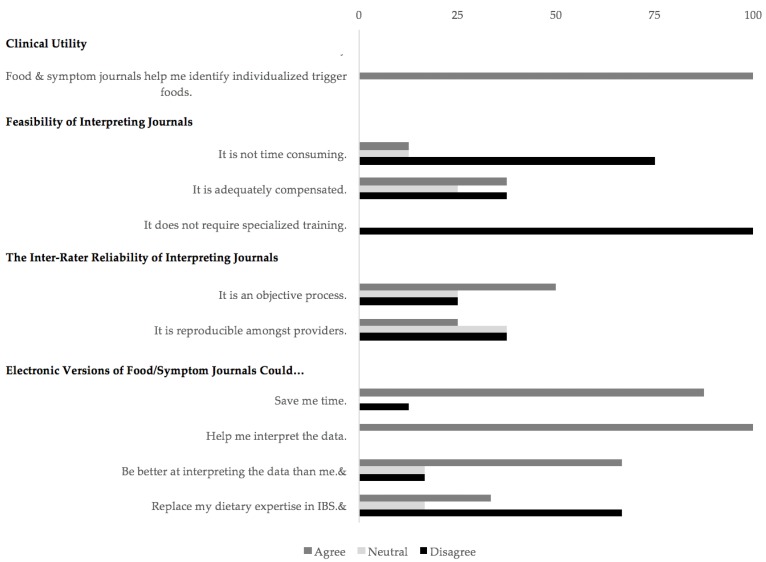
Intake questionnaire: provider perspectives on IBS food and symptom journals: utility, feasibility, inter-rater reliability, and electronic journals. *N* = 6 (all other responses, *N* = 8).

Providers completed an intake questionnaire. The intake questionnaire asked providers to agree or disagree (five-point Likert scale) on: (1) whether they considered certain food groups as potential IBS trigger foods (Figure 1), (2) statements on the utility, feasibility, and inter-rater reliability of IBS journals, and (3) statements on electronic journals.

### 2.2. Journal Interpretations

Providers completed a journal evaluation after reviewing each IBS patient’s set of journals. The journal evaluation asked providers to write down the dietary recommendation(s) s/he would most likely give to the patient. Second, it asked providers to rate the trigger food likelihood of key food groups (i.e., FODMAPs, fructose, lactose, fructans, galactans, polyols, high-calorie meals, high-fat foods, gluten, caffeine, high fiber, spicy foods, alcohol) for the patient (five-point Likert scale). Providers could select “not enough information”. Providers reviewed the 17 journals in the same sequence.

In the absence of a standardized instrument, K.C. and J.Z. developed the intake questionnaire and journal evaluation. A panel of experts in the field of questionnaire design reviewed the drafts for face validity and provided feedback. The revised instruments were further reviewed and edited by a clinical dietitian (J.S.) and gastroenterologist. Participating providers were compensated $20 for completing each journal evaluation.

### 2.3. Categorization of Written Recommendations

The written recommendations were categorized into different themes by J.Z., and J.S. confirmed these categorizations. Any disagreements were resolved by a discussion between J.Z. and J.S. If the rationale was not specifically spelled out, we did not categorize it into its broader possible categories. For example, if a provider wrote to “avoid onions”, we only categorized this recommendation as “avoid onions”. We did not categorize this recommendation also as “follow a low FODMAP diet” or “avoid fructans” (even though onions are known to be high in fructans, which is a type of FODMAP). However, if a provider *specifically* wrote “avoid onions due to fructans”, we categorized this recommendation as “avoid onions”, “follow a low FODMAP diet”, and “avoid fructans”.

Some exceptions were made to these guidelines. If “avoid cream” was written, we categorized this as “avoid lactose” and “avoid high fat”. If “avoid coffee” was written, we categorized this as “avoid caffeine” only. If a provider recommended to avoid multiple fruits or vegetables, we categorized these recommendations collectively as “avoid fruits” or “avoid vegetables”, respectively. However, if more than one provider recommended to avoid a specific fruit or vegetable, we included this recommendation into its own separate category such as “avoid apples”.

### 2.4. Statistical Analysis

The Krippendorff’s α-reliability estimate was used to determine the inter-rater agreement of trigger food ratings for all providers using the R Statistical Software v2.15.2 (R Foundation for Statistical Computing, Vienna, Austria) [13]. A separate estimate was performed for dietitians and medical providers only. One provider was both a trained dietitian and medical provider and included in both estimates. If a provider did not provide a trigger food rating or selected “not enough information” for a food group, we treated this as missing data. To describe the inter-rater agreement of written recommendations, we tallied the number of providers who wrote a similar recommendation for each patient.

To highlight each provider’s variability in his/her own trigger food ratings, we graphed each provider’s mean trigger food likelihood ratings versus his/her standard deviation of ratings for each food group. To describe the variability of each provider’s written recommendations, we collectively displayed every written recommendation an individual provider gave to our patients.

### 2.5. Qualitative Data

Providers were asked to “think aloud” on how they review an IBS journal. They were also asked whether they thought journaling was a valuable experience for them and their patients. During the exit interview, they were asked to describe their journal review practice and challenges. Emerging themes from these observations and responses were identified to reflect provider attitudes by C.C. J.Z. confirmed these emerging themes. Any disagreements were resolved by a discussion between J.Z. and C.C.

## 3. Results

### 3.1. Participating Providers

Eight out of 11 interested providers met our inclusion criteria and agreed to participate in our study. Our providers consisted of two gastroenterologists, one gastroenterologist/dietitian, one family medicine doctor, one nurse practitioner, and three dietitians. Of the excluded providers, two had no experience in reviewing journals and one failed to respond. Participating providers independently reviewed the 17 de-identified journals. Five providers practiced at the University of Washington Medical Center (Seattle, WA, USA); two providers were in private practice (Seattle, WA, USA and Boston, MA, USA). All but one provider was female. All participating providers had at least some training in the dietary management of IBS. Our providers had on average, 17.3 (range 6–30) clinical years of IBS experience. They saw approximately 20 (range 8–70) IBS patients and reviewed 10 (range 2–30) journals monthly.

### 3.2. Provider Perspectives

During the intake questionnaire prior to the study, all providers (*N* = 8) agreed that certain foods could cause IBS flare-ups and that these foods varied amongst patients. Figure 1 displays the average provider ratings on whether they considered certain food groups as potential IBS triggers. Figure 2 shows our provider perspectives on the clinical utility, feasibility, and inter-rater reliability of IBS journals and on electronic journal versions.

### 3.3. Inter-Rater Agreement of IBS Journal Interpretations

#### 3.3.1. Trigger Food Likelihood Ratings

Estimates of agreement of trigger food likelihood ratings amongst providers were poor, overall (average α = 0.07). Table 1 outlines the estimates of agreement among all providers, only dietitians, and only medical providers for each food group. Dietitians agreed fairly well on their assessment of alcohol (α = 0.3) as a potential trigger food. Medical providers agreed fairly well on their assessment of high-fat foods (α = 0.3) and polyols (α = 0.4) as potential trigger foods.

#### 3.3.2. Written Recommendations

Written recommendations varied widely. The average number of recommendation each provider wrote for a patient was 6 (range 2–14). When combining all providers, the average number of different recommendations given to a single patient was 27 (range 11–56). For every patient, there was at least one recommendation given by ≥4 providers, but most recommendations were given by ≤3 providers. Supplemental Table S1 summarizes the number of providers who gave similar written recommendations to each patient.

### 3.4. Variability of Provider Trigger Food Ratings and Written Recommendations

#### 3.4.1. Trigger Food Likelihood Ratings

Five providers demonstrated low variability in their trigger food likelihood ratings for at least half of the food groups. Supplemental Figure S1 includes graphs for each provider to highlight his/her variability in trigger food ratings. Provider 2 had the least amount of variability in his/her trigger food likelihood ratings. Provider 2’s standard deviations for all his/her trigger food likelihood ratings were ≤1.0. This translated to minimal discrimination in trigger food likelihood ratings despite individualized journal reviews. For example, Provider 2 rated “high fiber” as an “unlikely” trigger food group for 12 of the journals. S/he never rated “high fiber” as a “likely” or “highly likely” trigger food for any of the journals s/he reviewed. A similar pattern was observed for other food groups. Another extreme case was Provider 6. Provider 6 rated caffeine and spicy foods as a “highly unlikely” trigger food for all 17 journals.

#### 3.4.2. Written Recommendations

Four of our providers gave the exact same written recommendation(s) (range 3–7) to over half of the patients (Supplemental Table S2).

### 3.5. Qualitative Data

Most providers used other tools to support their interpretation. They broke down nutrients, tallied the number of occurrence of potential triggers, and wrote down their hypotheses on the journal or a separate sheet of paper (*N* = 7). Providers also used various strategies to analyze the data. Most providers read through the first few journal entries and developed initial hypotheses of potential triggers. Some then focused the rest of their review on verifying these hypotheses by skimming the rest of the journal entries. Others developed new hypotheses during the review process.

Providers found it difficult to manually correlate food and symptoms (*N* = 8). Some found journal interpretation difficult because they lacked the knowledge of detailed nutrient information for certain meals (*N* = 4). Some providers wanted additional information, such as stress levels (*N* = 3), baseline GI symptoms (*N* = 2), and non-GI symptoms (*N* = 2) to help them better understand a patient’s journal. Despite these challenges, most providers still considered journaling a valuable process as it improves provider-patient relationship (*N* = 8), holds patients accountable (*N* = 6), and increases patient awareness and knowledge (*N* = 4).

## 4. Discussion

The inter-rater reliability of provider interpretations of IBS food and symptom journals was poor, overall. Agreement of trigger food likelihood ratings amongst our providers was poor. There was minimal overlap in the written recommendations given to each IBS patient by our providers. Providers also seemed to favor certain trigger food likelihood ratings and written recommendations. Most providers gave similar trigger food likelihood ratings for at least half of the food groups. Half of the providers gave the exact same written recommendation(s) to over half the patients.

These results call into question the presumed clinical utility of IBS journals and the current variable methods used to identify personalized trigger foods from these journals. It emphasizes the need to standardize journaling practices and methods used to interpret such journals. This method can then be validated for the accurate identification of personalized trigger foods.

These findings are not surprising. Providers have variable methods on how they review and interpret these journals. This has been described in a study by Chung et al. who interviewed 21 IBS providers and again in this study [11]. Providers likely have such different methods of interpreting IBS journals because they receive variable education on the dietary management of IBS. This education is largely dependent one’s training institution, mentoring faculty, medicine specialty, and even generation.

Another strong influence is a provider’s past clinical experience with the dietary management of IBS. This likely explains why providers have “favorite” dietary recommendations. Providers may value certain exclusionary diet(s) and eating habit(s) over others because of their prior clinical success with those specific recommendation(s). This clinical success can include a reduction of IBS symptoms or sustained adherence to specific dietary recommendation(s).

Providers may also be biased towards certain recommendations because they feel more skilled and familiar with it. Due to this familiarity, providers may be better at recognizing certain trigger foods over others in a journal. Conversely, there may be some trigger foods providers are less familiar with and less able to recognize in a journal. This is one of the main challenges expressed by our providers during the exit interview.

At present, there have been limited studies exploring standardized methods for the identification of personalized trigger foods from food and symptom journals. In a study by Kueper et al., regression analyses were used to identify triggers from food and symptom journals in 164 patients with chronic medical problems [14]. Their analyses identified a unique set of trigger foods for most participants. When these analyses were used as a dietary guide for exclusionary diets, it helped reduce symptoms for 75% of the study participants. We have conducted similar exploratory analyses to highlight associations between meal nutrients and symptoms using the 17 journals in this study [12,15]. We have also explored the use of *N*-of-1 trials as an alternative method [16]. Further studies are needed to validate these proposed methods.

We have previously shown that journaling alone did not impact IBS symptoms, at least in the short-term [12,17]. This was a surprising finding given that journaling promotes the self-management of IBS. In both of these studies, participants stressed the need for more guidance and “answers” from their journaling efforts.

Current journaling methods may also explain the poor inter-rater reliability of providers’ interpretations of IBS journals. More rigorous techniques, such as trial diets (e.g., an elimination-challenge diet) may be needed to accurately identify personalized IBS food triggers. Attempts have been made to provide evidence-based guidelines in trial diets, but challenging given such different approaches and study outcomes [18]. Further research is clearly needed in this area

### Study Limitations

A major limitation of this study was the inability of our providers to interact with the patients who authored the journals. Providers were unable ask about other factors (e.g., stress, sleep) contributing to a patient’s symptoms. The therapeutic effect of patient-provider interaction was also completely removed from this study [19]. This could have significantly impacted our providers’ interpretation of these journals.

“Fatigue” from interpreting all 17 journals might have affected our providers’ journal interpretations. We were also unable to determine a providers’ competence in interpreting these journals. If a provider did not provide a trigger food rating or selected “not enough information,” we treated this as missing data. However, a patient’s journal might have truly lacked the data to conclude whether a specific food group was or was not a trigger food. Given that there is no “gold standard” on how to interpret these journals, we were unable to measure whether fatigue or incompetence affected our providers’ journal interpretations. If such measures were possible and did indeed demonstrate fatigue and/or incompetence in our providers’ journal interpretations, this would emphasize the need for more training rather than a change in the current journaling practices.

Our methodology in collecting our providers’ written recommendations might not have accurately captured our providers’ true journal interpretation. We assumed the rationale for the written recommendations was solely based on the patient journal reviewed. However, this might not be the case. Providers might have a standard set of initial dietary recommendations to give to all their IBS patients. Some exclusionary diets might be easier for patients to identify and eliminate from their diet than others. Providers might have different knowledge (and, thus, comfort) levels in educating patients about certain exclusionary diets and/or dietary habits over others. Finally, certain dietary habits might be easier to integrate into a busy patient’s life than others.

Our categorization of written recommendations might have also underestimated the true inter-rater reliability of our providers’ journal interpretations. If the rationale behind a written recommendation was not clearly spelled out, we did not categorize it into its broader possible categories. For example, if a provider wrote to “avoid granola bars”, we did not categorize this recommendation into any of the following broader categories, such as “avoid high fructose”, “avoid gluten”, etc. We cannot exclude the possibility that this was, in fact, the true rationale behind a written recommendation.

Finally, these study results may not accurately reflect the perspectives or journal interpretations of all IBS providers. Most of our providers practiced at a single academic institution and were female.

## 5. Conclusions

In conclusion, the inter-rater reliability of provider interpretations of IBS food and symptom journals was overall poor. Providers seemed to favor certain exclusionary diets and dietary recommendations over others. This supports the need for a more standardized method for interpreting these journals (e.g., regression analyses) and/or more rigorous techniques (e.g., *N*-of-1 trials, elimination-challenge diet) to accurately identify personalized IBS food triggers.

## Figures and Tables

**Table 1:** Inter-rater agreement of trigger food likelihood ratings amongst providers after reviewing 17 IBS food and symptom journals using Krippendorff α-reliability estimates.

Food Groups	All Providers *N* = 8		Dietitians *N* = 4		Medical Providers *N* = 5	
	α	95% CI	α	95% CI	α	95% CI
FODMAPs	0.0	−0.1, 0.2	0.0	−0.2, 0.2	0.1	−0.2, 0.4
Fructose	0.1	0.0, 0.3	0.0	−0.2, 0.2	0.1	−0.2, 0.4
Lactose	0.1	−0.1, 0.2	0.1	−0.2, 0.3	0.1	−0.1, 0.4
Fructans	0.0	−0.2, 0.2	0.0	−0.2, 0.2	0.0	−0.2, 0.3
Galactans	0.0	−0.1, 0.1	0.0	−0.2, 0.2	0.0	−0.4, 0.4
Polyols	0.1	−0.1, 0.3	0.1	−0.2, 0.4	0.4	−0.2, 0.7
High-calorie meals	0.1	0.0, 0.3	0.1	−0.1, 0.3	0.2	−0.1, 0.3
High-fat foods	0.2	0.0, 0.3	0.2	−0.1, 0.3	0.3	0.1, 0.4
Gluten	−0.1	−0.1, 0.0	−0.1	−0.3, 0.1	−0.2	−0.3, −0.1
Caffeine	0.0	−0.1, 0.1	0.2	−0.1, 0.4	0.0	−0.1, 0.1
High Fiber	−0.1	−0.1, 0.0	0.0	−0.2, 0.2	−0.1	−0.2, 0.0
Spicy Foods	0.1	−0.1, 0.2	0.0	−0.2, 0.2	0.0	−0.2, 0.2
Alcohol	0.1	0.0, 0.2	0.3	−0.1, 0.5	0.0	−0.2, 0.1

IBS = irritable bowel syndrome; FODMAPs = fermentable oligo-di-monosaccharides and polyols. Unshaded cells: poor agreement (α ≤ 0.20). Shaded cells: at least fair agreement (α: 0.21–0.40). One provider was both a dietitian and medical provider. If a provider did not provide a trigger food rating or selected “not enough information” for a food group, this was treated as missing data.

## Supplementary Materials

The following are available online at www.mdpi.com/2077-0383/6/11/105/s1; Figure S1: Variability of food trigger ratings for each provider: trigger food likelihood journal rating means versus standard deviation for each main food group; Table S1: Provider variability of written recommendations: number of providers who gave similar written recommendation(s) to each participant; Table S2: Provider variability of written recommendations.
