# Supplementary material for: Inter-Rater Reliability of Provider Interpretations of Irritable Bowel Syndrome Food and Symptom Journals
Source: J Clin Med. 2017 Nov 4;6(11):105. doi: 10.3390/jcm6110105 (PMC5704122; doi:10.3390/jcm6110105)
Supplement: Supplementary file 1 [file jcm-06-00105-s001.pdf]

Supplemental Materials

Supplemental Figure 1. Did providers vary their trigger food likelihood ratings amongst the 17 IBS food and symptom journals? Plots of each provider's average trigger food likelihood ratings versus standard deviations for each main food group.

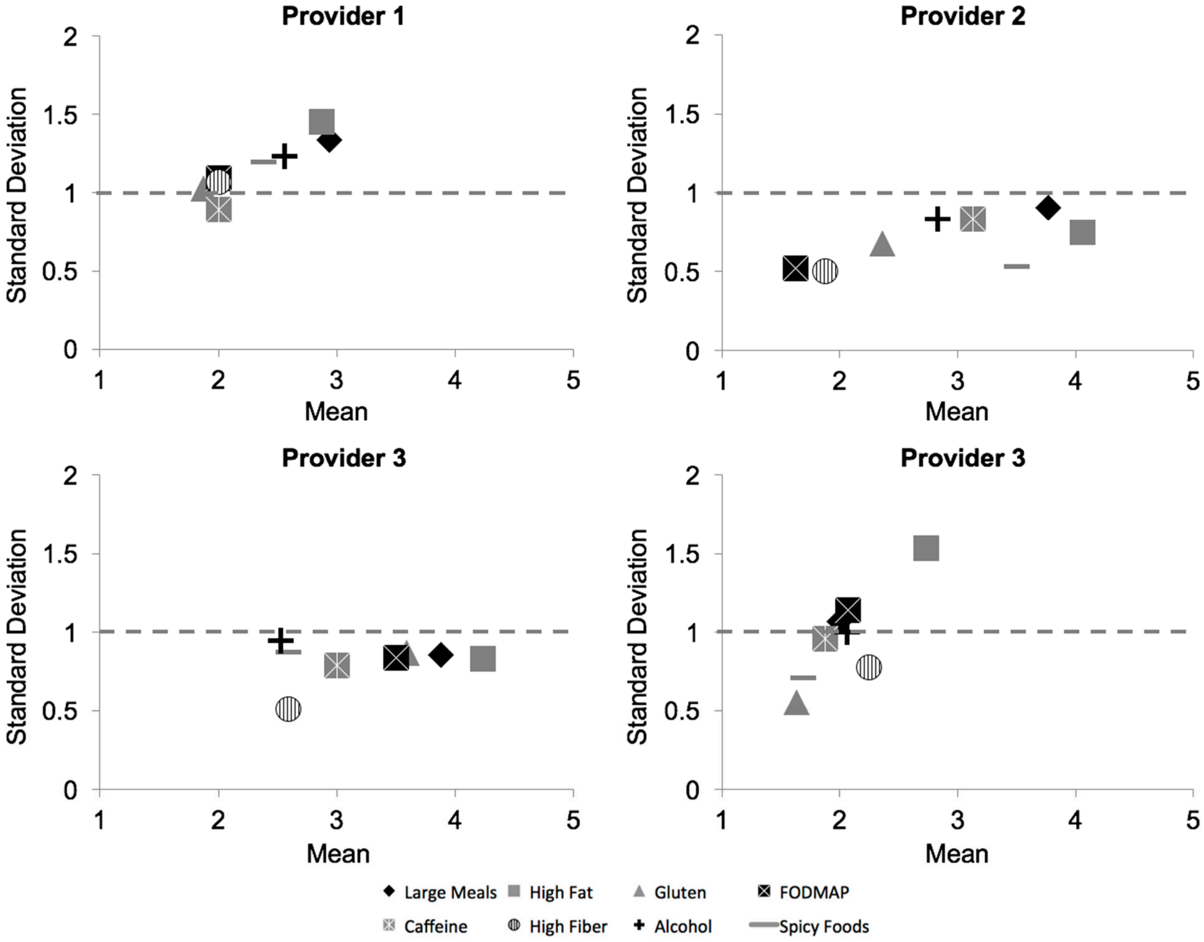

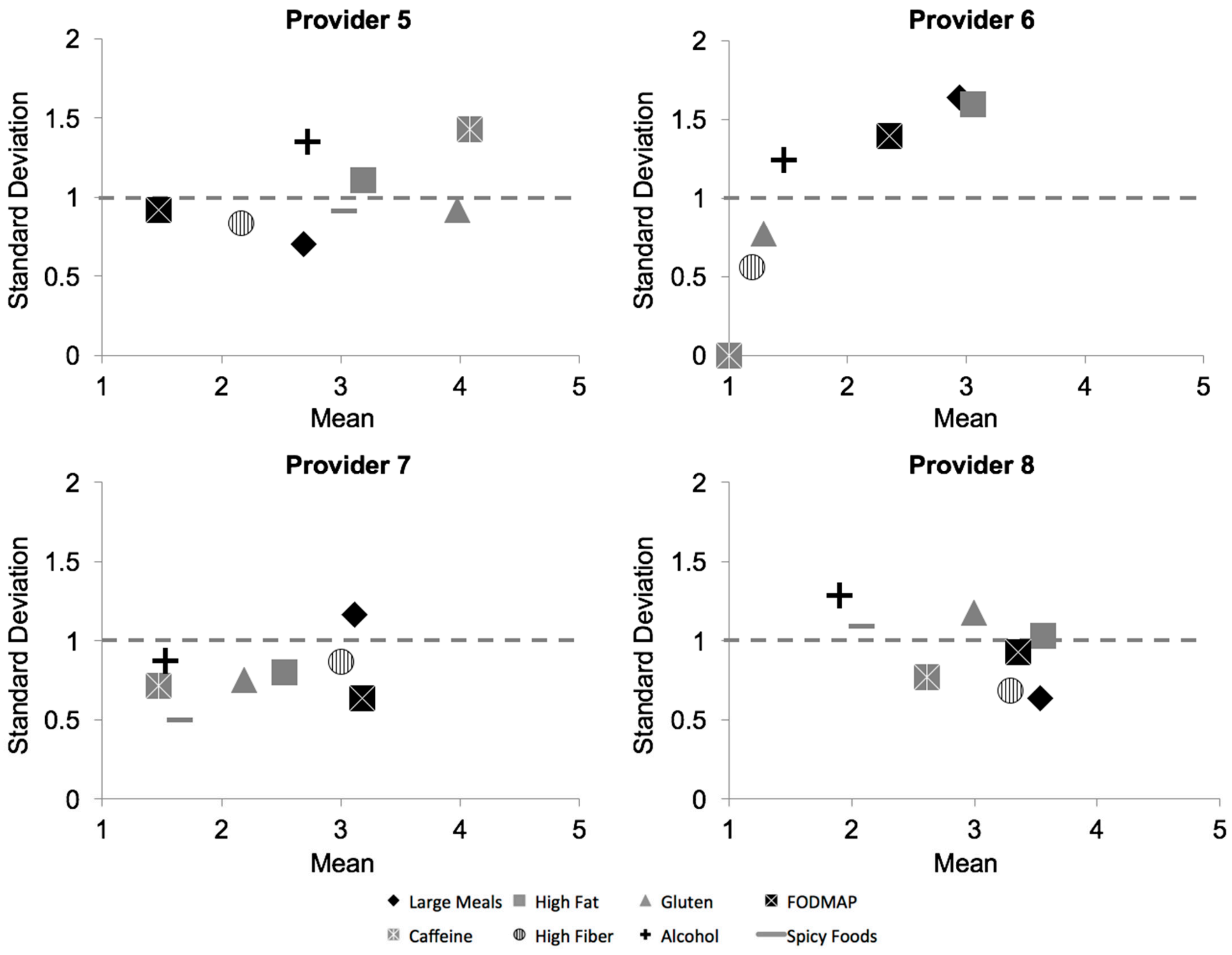

FODMAP = fermentable oligo-di-monosaccharides and polyols

Supplemental Table 1. Provider variability of written recommendations: Number of providers who gave similar written recommendation(s) to each participant.

	Participant																
**Written Recommendations**	2	3	4	5	6	7	8	9	10	11	12	13	14	15	16	17	18
**Meal Timing/Pattern**																	
Avoid eating before bedtime	2					1	2						1	1		1	1
Eat breakfast	2				1	1	1	1		1	1		2	1			1
Eat more frequently			3	2	2	3	3	3	2		3		2	3			
Eat regular meals	3	1								2		3			4	3	4
Smaller meals		1		3	1		1	1	4	2	1		2	2		2	1
**Specific Diets**																	
Anti-inflammatory diet												1			1		
Elimination Diet	2					1											
**Exclusionary Diets**																	
Avoid alcohol				1			1			2	1			6			
Avoid beer									3							2	3
Avoid caffeine	2			1	1		4	1	2	2	2	1	1	2		3	3
Avoid cereal		2								1						1	
Avoid cheese						2				2							
Avoid chicory root				1													
Avoid chocolate					1	1	1					1					2
Avoid curry		1											1				
Avoid dried fruit											1						
Avoid dates		2															
Avoid prunes							2										
Avoid eggs						1		2					1		1		1
Avoid fiber	1																

Written Recommendations	Participant																
	2	3	4	5	6	7	8	9	10	11	12	13	14	15	16	17	18
Avoid FODMAPs	4	5	3	3	4	3	6	2	3	2	4	4	2	4	4	3	5
Avoid fructans				1		3						1	1	1			1
Avoid fructose	1	1	2	2	3	3	2	1	1	1		2	1	1		1	1
Avoid galactans				1		1								3			1
Avoid inulin			2														
Avoid lactose	1	1	1	4	2	1	1	1	4	3	2	2	2	1	3	5	1
Avoid oligosaccharides			1						1								
Avoid polyols								1				1	1			1	
Avoid fresh veggies			2		1		3				2			1		1	1
Avoid cruciferous vegetables				2			2		2								
Avoid beans		2		2	1	1	2	2	2					3	1		2
Avoid cabbage								2									
Avoid carrots								1									
Avoid corn			1														
Avoid garlic							1										
Avoid kale																	2
Avoid lentils									2								
Avoid mushrooms		1	2						1								
Avoid onions		1					1							3			
Avoid peas								1					2				
Avoid fruits		2	1		3	2	3		3	4		1	6	4			
Avoid apples					2		4			2	1			3			3
Avoid avocados							2		2								
Avoid bananas													1		1		
Avoid citrus fruit													3				
Avoid fruit juices	1																
Avoid pears									2	3							3

Written Recommendations	Participant																
	2	3	4	5	6	7	8	9	10	11	12	13	14	15	16	17	18
Avoid gluten	1	4	1	1		1	1	1			1	2		1	2	1	1
Avoid ham	1																
Avoid high fat foods	6		5	4	1	1		4	4	5	4	2	2	2	5	5	3
Avoid honey							2						3	1			
Avoid hummus																1	
Avoid large meals	2		4			5						2			4		
Avoid nuts			1			1				1	1	1	4		2		2
Avoid peanut butter									1	1							
Avoid popcorn																	2
Avoid prepared foods							1										
Avoid processed foods	1										1			1	3	1	
Avoid protein bars		2	2		2												2
Avoid red meats										3			1				
Avoid soda								2							1	1	
Avoid carbonation	1	1			2												
Avoid Diet Coke														2			
Avoid soy			3			1	2	2		1		4		3			
Avoid spicy foods				1				2		2			2	2	1		
Avoid sugars			1		2		2		2			4		2	1	1	1
Avoid tea										1							
Avoid chamomile tea					1						1						
Avoid wheat		3	2	1			1	1	2	1		1				4	
**Meal Preparation**																	
All liquid breakfast													1				
Avoid coffee alone											1					1	
Cook vegetables				2	1											1	1
**Consume more…**																	
Calories					1												1
Fiber	1		1	4		2	2	2	2	2	2	2	1	3	3	2	2

**Written Recommendations**	**Participant**																
	2	3	4	5	6	7	8	9	10	11	12	13	14	15	16	17	18
Fluids	1	1	1	2	1	2	2	1	1	1	1	1	1	2		2	2
Fruits/vegetables					1	1									1	2	1
Grains					1												
Protein					1				1			1			1	1	2
**No relationship found**																	
No relationship found	1	1			1	1	1	2		1			1				
**Total # of written recommendations given by all providers**	18	18	20	19	24	22	27	22	22	24	18	20	24	25	18	23	29

FODMAP = fermentable oligo-di-monosaccharides and polyols

Highlighted cells represent ≥ 4 providers wrote a similar written recommendation.

Empty cells signify that the participant did not receive the specific written recommendation by any provider.

Supplemental Table 2. Provider variability of written diet recommendations

Provider[&]	Years of IBS Clinical Experience	Written Recommendations Per Participant (mean + SD)	Total # of Different Written Recommendations for all Participants	Written Diet Recommendations	
				Diet Recommendation	# of Participants this Recommendation was given to (N, %)
1 (M, D)	6	2.5 ± 1.3	19	Avoid high fat foods	5, 29.4%
				Avoid large meals<br>Low FODMAP diet	4, 23.5%
				Avoid processed foods<br>Eat more regular meals<br>Increase fiber<br>Avoid lactose	3, 17.6%
				Avoid gluten<br>Decrease nuts	2, 11.8%
				Avoid spices<br>Substitute soda for water<br>Anti-inflammatory diet<br>Avoid sweets<br>Avoid bananas<br>Avoid eggs<br>Avoid beans/galactans<br>Eat more<br>Increase protein<br>Increase fruits/vegetables	1, 5.9%
2 (M)	20	13.5 ± 4.1	53	Eat smaller meals	17, 100%
				Avoid high-fat foods	15, 88.2%
				Increase fiber	13, 76.5%
				Eat more frequent meals	12, 70.6%
				Eat breakfast<br>Avoid high fructose foods<br>Low FODMAP	11, 64.7%
				Avoid raw vegetables<br>Eliminate apples	7, 41.2%
				Avoid lactose<br>Increase water intake<br>Reduce sugar	6, 35.3%
				Avoid diet sodas<br>Avoid caffeine	5, 29.4%

				Avoid spicy foods	
				Avoid gluten<br>Avoid polyols	4, 23.5%
				Avoid processed foods<br>Avoid salads<br>Avoid galactans<br>Eat oatmeal for breakfast<br>Increase fruits/vegetables<br>Avoid pears<br>Avoid beans<br>Avoid beer<br>Avoid red meats	3, 17.6%
				Add protein<br>Avoid cheese<br>Eat an earlier dinner<br>Avoid galactans<br>Avoid curry<br>Eliminate dates<br>Eliminate cereal<br>Avoid fruits<br>Avoid protein powder	2, 11.8%
				Eliminate mushrooms<br>Eliminate carbonation<br>Reduce protein<br>Eliminate blackberries<br>Decrease prepared foods<br>Avoid avocados<br>Avoid fructans<br>Avoid liquid breakfast<br>Avoid oranges alone<br>Avoid diet coke<br>Avoid coffee alone<br>Avoid chocolate<br>Avoid popcorn<br>Add low-FODMAP fiber	1, 5.9%
3 (M)	20	5.8 ± 2.0	35	Eat regular meals	9, 52.9%
				Avoid high-fat foods	8, 47.1%
				Avoid eating before bed	7, 41.2%
				Avoid wheat<br>Avoid lactose	6, 35.3%

				Decrease fructose<br>Avoid soy<br>Eat smaller meals<br>Avoid sugar	4, 23.5%
				Low FODMAP diet<br>Elimination diet<br>Eat breakfast<br>Avoid cruciferous foods<br>Avoid eggs<br>Avoid caffeine	3, 17.6%
				Avoid gluten<br>Avoid inulin<br>Avoid apples<br>Avoid avocados<br>Avoid nuts<br>Avoid diet soda	2, 11.8%
				Avoid beans<br>Avoid kind bars<br>Avoid corn<br>Avoid mushrooms<br>Cook vegetables<br>Avoid peas<br>Avoid carrots<br>Avoid meats<br>Avoid oranges<br>Avoid citrus foods<br>Avoid alcohol<br>Avoid spicy foods<br>No clear relationship	1, 5.9%
4 (D)	20	6.3 ± 2.6	37	Avoid lactose<br>Avoid fruits<br>Avoid beans	8, 47.1%
				Avoid raw vegetables	7, 41.2%
				Avoid greasy/fatty foods<br>Avoid soy	6, 35.3%
				Reduce protein bars	5, 29.4%
				Avoid alcohol<br>Avoid chamomile tea<br>Avoid fructans<br>Avoid fructose	4, 23.5%

				Avoid coffee<br>Reduce wheat<br>Avoid inulin<br>Avoid kind bars<br>Avoid peanut butter	3, 17.6%
				Avoid FODMAPs<br>Avoid carbonation<br>Avoid mushrooms<br>Smaller meals<br>Avoid sugars<br>Increase protein<br>Avoid nuts	2, 11.8%
				Elimination diet<br>Reduce dates<br>Avoid avocados<br>Avoid prunes<br>Avoid meats<br>Avoid dried fruits<br>Eat something with coffee<br>Reduce yogurt<br>Reduce juices<br>Avoid spicy foods<br>Reduce carbohydrates<br>Avoid apples<br>Avoid galactans<br>Avoid pears	1, 5.9%
5 (M)	20	5.8 ± 2.5	20	Avoid caffeine	12, 70.6%
				Decrease lactose	10, 58.8%
				Avoid gluten<br>Reduce wheat<br>Reduce fruits (citrus)	9, 52.9%
				Avoid high fat foods<br>Avoid spicy foods<br>Avoid chocolate	6, 35.3%
				Low FODMAP diet<br>Reduce fructans<br>Reduce alcohol<br>Avoid lactose<br>Avoid soy<br>No clear relationship	4, 23.5%

				Eat smaller meals<br>Reduce eggs	2, 11.8%
				Avoid nuts<br>Reduce fructose<br>Avoid beans<br>Avoid onions	1, 5.9%
6 (M)	30	2.1 ± 0.9	10	Avoid high-fat foods	8, 47.1%
				Low FODMAP diet	7, 41.2%
				Not related to foods	6, 35.3%
				Eat smaller meals	5, 28.4%
				Avoid alcohol	3, 17.6%
				Avoid gluten<br>Increase fiber<br>Avoid caffeine<br>Avoid sweets<br>Avoid cheerios	2, 11.8%
7 (D)	7	4.6 ± 0.9	15	Increase water intake<br>Regular meal pattern<br>Low FODMAP<br>Increase fiber	17, 100%<br>16, 94.1%<br>16, 94.1%<br>16, 94.1%
				Reduce sugars<br>Decrease fruits<br>Eat more vegetables/fruit	2, 11.8%
				Increase calories<br>Increase protein<br>Substitute sugars<br>Decrease nuts<br>Avoid alcohol<br>Avoid processed foods<br>Reduce wheat/gluten<br>Decrease apples	1, 5.9%
8 (D)	15	3.2 ± 1.6	15	Low FODMAP	13, 76.5%
				Avoid fructose	11, 64.7%
				Avoid high fat foods	9, 52.9%
				Avoid oligosaccharides	5, 29.4%
				Avoid alcohol<br>Avoid fructans	3, 17.6%
				Avoid caffeine<br>Avoid gluten	2, 11.8%

Avoid high-fiber foods	
Avoid soy	
Avoid lactose	
Avoid nuts	1, 5.9%
Eat smaller meals	
Avoid honey	
No consistent pattern	

FODMAP = fermentable oligo-di-monosaccharides and polyols

&Type of Provider (M = Medical Provider; D = Dietitian
